# Direct synthesis of nanodiamonds by femtosecond laser irradiation of ethanol

**DOI:** 10.1038/srep33966

**Published:** 2016-09-23

**Authors:** Chen-Hon Nee, Seong-Ling Yap, Teck-Yong Tou, Huan-Cheng Chang, Seong- Shan Yap

**Affiliations:** 1 Faculty of Engineering, Multimedia University, 63100 Cyberjaya, Selangor, Malaysia; 2 Department of Physics, Faculty of Science, University of Malaya, 50603 Kuala Lumpur, Malaysia; 3 Institute of Atomic and Molecular Sciences, Academia Sinica, Taipei 106, Taiwan

## Abstract

Carbon nanomaterials exhibit novel characteristics including enhanced thermal, electrical, mechanical, and biological properties. Nanodiamonds; first discovered in meteorites are found to be biocompatible, non-toxic and have distinct optical properties. Here we show that nanodiamonds with the size of <5 nm are formed directly from ethanol via 1025 nm femtosecond laser irradiation. The absorption of laser energy by ethanol increased non-linearly above 100 μJ accompanied by a white light continuum arises from fs laser filamentation. At laser energy higher than 300 μJ, emission spectra of C, O and H in the plasma were detected, indicating the dissociation of C_2_H_5_OH. Nucleation of the carbon species in the confined plasma within the laser filaments leads to the formation of nanodiamonds. The energy dependence and the roles of the nonlinear phenomenon to the formation of homogeneous nanodiamonds are discussed. This work brings new possibility for bottom-up nanomaterials synthesis based on nano and ultrafast laser physics.

Nanodiamonds/diamond nanoparticles are formed by fully sp^3^/tetrahedral hybridization, with a diameter of <100 nm. Nanodiamond was first discovered in primitive meteorites^1^; the formation environment whether it was in a pre-solar or inner solar nebula is important to astrophysical research^2^. Single digit nanodiamonds (<5 nm, with about 1600 carbon atoms), are of high demand to many biomedical applications^3^ especially as biomarker^4^ where the presence of nitrogen vacancy (NV) centers in nanodiamonds results in the absorption of the visible spectra and emission in red at room temperature with great temporal stability and almost no photobleaching^5^,^6^. A single nanodiamond crystal is capable of producing luminescence brighter than standard fluorescent protein. This is demonstrated in a recent study where the fluorescence of nanodiamonds greatly surpassed the conventional dye used in cancer cell detection^3^ while another research indicates that the efficiency of drugs-delivery to tumor/cancer cell with nanodiamonds increases in cancer treatment, along with lower side-effect^7^. Recently, nanodiamonds with silicon vacancy have also been shown to produce stable near infrared fluorescence^8^. However, the sample was prepared with nanodiamonds from meteorites.

Nanodiamond growth is currently limited to detonation technique^9^ and chemical vapour deposition (CVD)^10^. Detonation of TNT and RDX explosives normally produced detonation nanodiamonds (DND) that varied in sized, purity, and phase (allotropes). Purification is needed and high energy proton beam irradiation, followed by annealing >700 °C to introduce vacancy in a post-growth process. CVD is also used to produced crystalline diamond in the form of films but require high substrate temperature and carbide forming substrates. On the other hand, pure physical growth of nanoparticles has recently been explored by pulsed laser ablation in liquid (PLAL), performed by laser ablation onto a target submerged in liquid. Laser plume interaction and collisional cooling occur while confinement of plasma plume by the medium promotes formation of crystalline nanomaterials^11^,^12^,^13^,^14^,^15^,^16^,^17^. Shockwave and quenching process maybe involved, creating an intrinsically favoured condition for nanodiamond formation. According to the growth kinetics model, nanodiamonds of 25–250 nm can be obtained by laser ablation of graphite in water^18^, close to the values obtained in PLAL of graphite experimentally^19^. In another approach, fermtosecond laser was used for dispersing and irradiation of carbon powder. Upon irradiation, particles with various sizes were produced and centrifugation separated the nanodiamonds in the supernatant^20^. The process of irradiation/dispersion is also capable of removing the layer of amorphous carbon from the inner sp^3^ hybridized diamond particles^21^. Nevertheless, purification is needed as an additional step in these techniques.

To dates, there is yet a consistent and controlled method in producing homogeneous nanodiamonds. In this work, we report that ultrafast laser filamentation could be a better way to produce homogeneous nanodiamonds conveniently and consistently. Instead of top-down approach in nanomaterials synthesis such as detonation or PLAL, homogeneous nanodiamonds were produced directly from ethanol by ultrafast laser filamentation^22^, where the intense ultrashort laser pulses undergo nonlinear propagation in transparent media. This results in nonlinear optical processes of self-focusing, supercontinuum generation, intensity clamping and tunnel ionization of the surround medium^23^.

## Results

### Fs laser irradiation in ethanol

**Figure 1:**
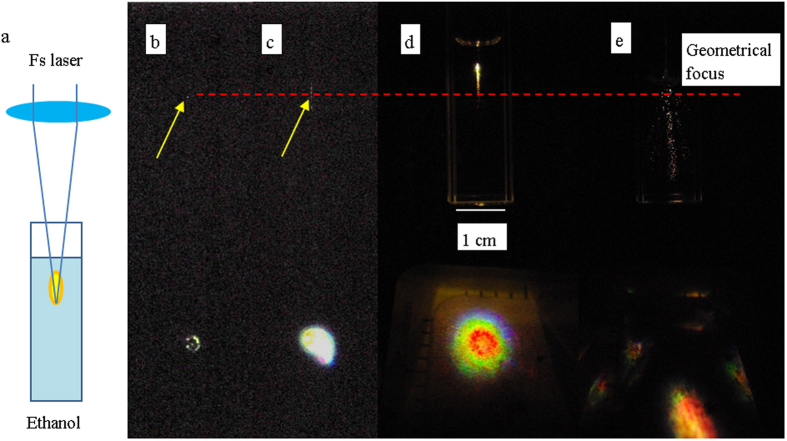
(**a**) Fs laser irradiation of ethanol. Side view images of pulsed fs laser irradiation of ethanol with incident laser energy of (**b**) 141 μJ, (**c**) 262 μJ, (**d**) 410 μJ and (**e**) 626 μJ. The emission spots are shown by the arrows. The geometrical focus of the laser beam is indicated by the dotted line. Images of pulsed fs laser irradiation of ethanol at 1 KHz repetition rate were taken with shutter speed of 1/1000 s.

In this work, 500 fs, 1025 nm laser pulses at 1 kHz repetition rate was focused into ethanol in a quartz cuvette. The experimental arrangement is shown in Fig. 1a and the experimental detail is given in the method summary. The critical power for self-focusing for ethanol, calculated based on the equation P_critial_ = 3.77λ^2^/8πn_0_n_2_, is ~1.5 × 10^6^ W with λ = 1025 nm, n_0_ = 1.355 and n_2_ = 7.7 × 10^−16^ cm^2^/W^24^. Varying the laser energy from 10 μJ to 650 μJ produced laser power of 10^7^–10^9^ W, about 1–3 orders of magnitude higher than the critical power for self-focusing in ethanol. Figure 1b–e show the side view of fs laser irradiation captured by a CCD camera with laser energy of 180 to 626 μJ. As the laser energy increased, filaments were observed before the geometrical focus of the laser beam, generating a streak along the laser axis, and transmitted through the bottom of the quartz cell.

**Figure 2:**
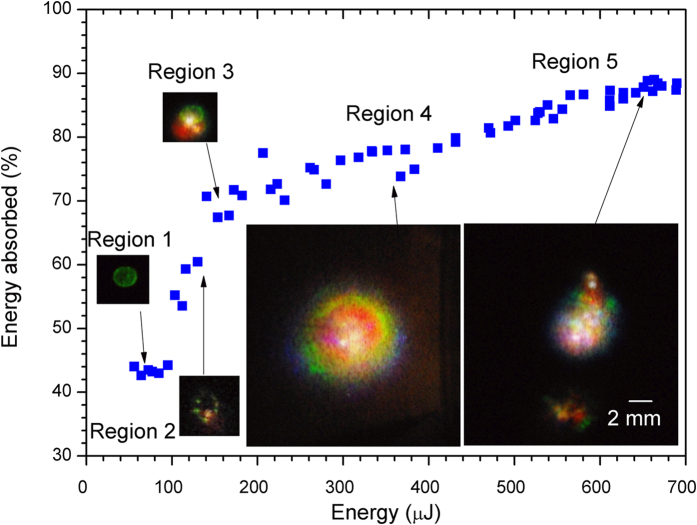
Absorption of fs laser pulse by ethanol. Energy absorbed at an incident laser energy of 50 to 680 μJ. In region 1, the absorption of the laser beam was contstant at ~43%. In region 2, absorption of laser energy increased sharply and filamentation began where color spots were detected in addition to the transmitted laser beam. In region 3, the absorbed energy increase slowly to ~75%. In region 4, the absorbed laser energy was stable at ~75–85%, and red, green and blue emissions were observed. In region 4, the absorbed energy increased to the maximum of ~90%, but the round red green blue beam was replaced by scattered emissions. The scale is the same for all the images.

The percentage of absorbed laser energy changed with the incident laser energy (E_i_) nonlinearly. The dependence of the percentage of absorbed energy on E_i_ is shown in Fig. 2, and can be divided into 5 regions:

1. Region 1, E_i_ < 100 μJ. The percentage of absorption remain constant at about 43%, independent of the incident laser energy due to linear absorption where he absorption coefficient ethanol at 1025 nm is ~0.13 cm^−1^, measured by using an unfocused laser beam. The value is close to the reported value of 0.138 cm^−1 ^25. The fs laser pulses focused and defocused in the solution; and the transmitted beam pattern was about 2 mm in diameter visible only on an IR viewing card. This indicated that there was no supercontinuum generation into the visible wavelength region; i.e. there was no filamentation^22^,^26^,^27^.
2. Region 2, 100 μJ < E_i_ < 150 μJ. As the incident energy increased from 100 μJ to 150 μJ. The percentage of energy absorbed increases very rapidly from 43% to 65%. Single bright emission spot surrounded by weaker spots was detected within the transmitted laser beam. This is an indication of the beginning of supercontinuum generation; i.e. filamentation^22^,^26^,^27^. The multiple spots indicated that a few filaments were formed. From the side view, emissions occurred at the geometrical focus of the laser beam, which indicates that laser induced breakdown also occurred (Fig. 1b)^28^.
3. Region 3, 150 μJ < E_i_ < 300 μJ. The percentage absorption of the incident laser energy were fluctuating around 65% to 75%. Filamentary spots of red, green and blue spots could be identified around the outer ring of the laser beam, while the central part observed was a bright white spot. This was an indication of multiple filamentation^22^,^26^,^27^. However, because there is a strong linear absorption, the laser’s peak power has to be much stronger than that in a non-absorbing medium. Indeed, the laser power was about 100 times higher than the critical power for self-focusing (P_critial_). From the side view, bright spots were observed at and near the geometrical focus of the laser beam (Fig. 1c) indicating simultaneous breakdown^28^.
4. Region 4, 300 μJ < E_i_ < 530 μJ. The absorbed energy increased very slowly from about 75% to 85%. The resulted pattern of the laser beam consists of rings; red in the inner ring, followed by green and blue in the outer rings. Multiple white spots were observed at or around the centre of the beam. The cross section of the pattern in this region is largest. The projected diameter (~13 mm) was 6 times larger as compared to those obtained in region 1. In this region, the laser filament in ethanol appeared as a channel of bright ‘streak’ seen from the side, with the streak having a length of 9 mm, at a distance of about 6 mm before the geometrical focus (Fig. 1d).
5. Region 5, 540 μJ < E_i_. As the laser energy increased to above 540 μJ, violent and explosive emissions occurred. At this elevated energy level, ethanol was vaporized. Red, green blue emissions were no longer confined to the centre region (with reference to the fs laser beam), but scattered with large angle. The percentage of absorbed energy increased to >85%. From the side view, the streak is not localized, and colour spots axis was observed along a distance of about 20 mm. The emission along the laser axis is very dynamic, scattered to all directions (Fig. 1e).

**Figure 3:**
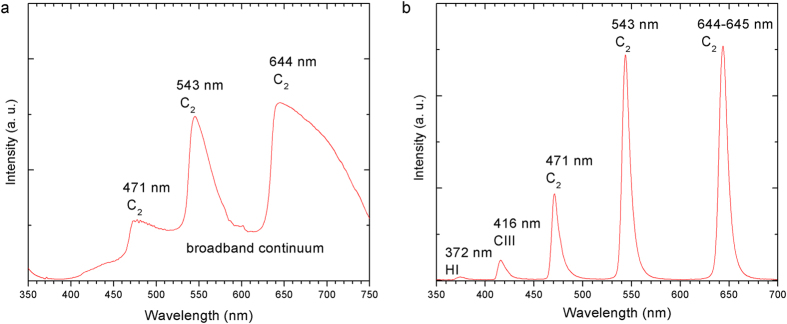
Optical emission spectra captured during laser irradiation of ethanol at 460 μJ. (**a**) At the forward direction and (**b**) from the side.

Figure 3a,b shows the optical emission spectra obtained at 460 μJ during laser irradiation. A white light continuum was generated as a results of filamentation in the forward direction (Fig. 3a), which has also been observed in various media^27^. The optical emission spectra of the streaks, was also measured from the side by imaging the streak with a ratio of 5:1 onto a fiber connected to a spectrometer. Sharp and prominent peaks at 644 nm, 543 nm, 471 nm for C_2_, 418 nm CIII and 372 nm HI were obtained (Fig. 3b), indicating the dissociation of ethanol. The peaks were identified by referring to the reported values and atomic spectra database^29^,^30^,^31^.

### Nanodiamond formation

**Figure 4:**
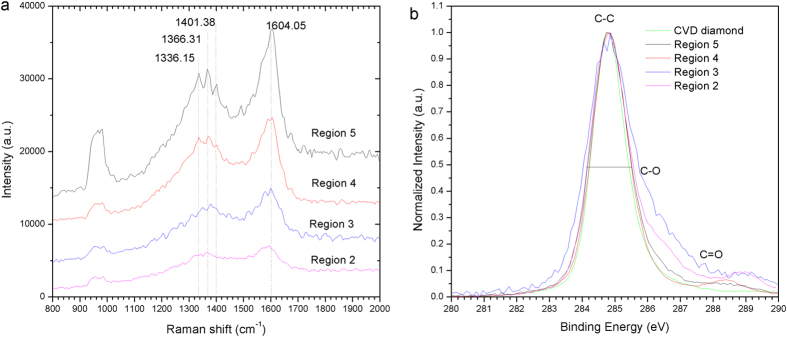
Raman and XPS spectra of the dried samples on Si substrate. (**a**) Raman spectra of nanodiamond samples grown at different incident laser energy. (**b**) XPS spectra of the prepared sample as compared to CVD diamond.

After fs laser irradiation, drops of the solution were casted onto pre-cleaned Si wafer and air-dried. The Raman spectra of the dried samples are shown in Fig. 4a. All the samples deposited at 120 μJ to 620 μJ exhibit the spectral characteristics of nanocrystalline diamond where a broadened peak at 1300 to 1380 cm^−1^ is observed. The spectra are comparable to those obtained in nanodiamond generated from microplasma dissociation of ethanol^32^. The asymmetrically broadened Raman spectra are observed because of the confinement of optical phonons in nanocrystals <10 nm, according to the phonon confinement theory^33^.

The spectra from X-ray photoelectron spectroscopy (XPS) of the samples obtained in region 2, 3, 4, 5 are compared to a CVD diamond sample in Fig. 4b. The method has been useful in detection of nanodiamonds as well as monitoring of the phase change of nanodiamonds upon thermal treatment^34^. The atomic concentrations of samples are typically: C (21.89%), O (40.93%), Si (37.19%) in a survey scan. The spectra are normalized in intensity and the peak position coinciding with the CVD diamond peak. The FWHM of the nanodiamonds obtained in this experiment were ~1.4 to 1.8 eV, while the FWHM of CVD diamond was ~1.3 eV. Sample grown at region 4 has the smallest FWHM which is closest to the CVD diamond spectra except that there are low intensity peaks at higher binding energy. The additional low intensity peaks at ~286 and ~289 eV corresponds to C-O and C=O bonds which were presence in the nanodiamond samples prepared in this work because of the synthesis process in liquid medium^20^,^35^.

**Figure 5:**
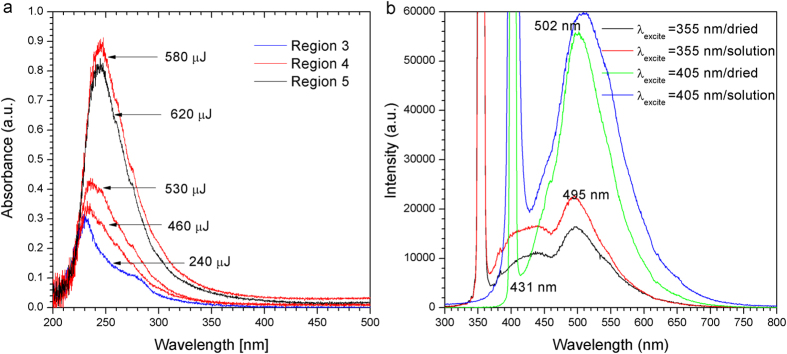
Absorption and flourescence spectra of ethanol solution after laser irradiation. (**a**) The absorbance is proportional to the incident laser energy until a saturation in Region 5. (**b**) Comparison of the photoluminescence sepctra of nanodiamond samples suspension and dried sample excited by 355 nm and 405 nm lasers.

Upon laser irradiation for 60 minutes, a change in the absorbance of ethanol solution was also observed. The absorbance spectra of the irradiated ethanol with reference to ethanol prior to irradiation are shown in Fig. 5a. The absorption peaks were detected at 225 nm to 260 nm, slightly red shifted with the increase of the laser energy. The absorbance intensity increased with laser energy until saturation at 580 μJ. In another work where nanodiamond was produced by the fs laser ablation of carbon powder in liquid, a peak between 228–238 nm was reported to be originated from the intrinsic absorption of nanodiamonds (σ-σ* transition, 228 nm) and π-π* transition of the C=C bond^20^. It is noted that multiple peaks in a single spectra (218 nm to 297 nm) have been observed when nanodiamond was produced from monocrystalline diamond and detonation which were processed by laser ablation in alcohol and underwent purification^36^. In that report, the observed peaks were suggested to have originated from a collection of multiple transitions and multiple polyaromatic chromophores from the sample produced from micron-sized diamond or detonation nanodiamond.

Blue to green fluorescence was obtained for samples suspended in ethanol solution and dried sample on Si substrates (Fig. 5b). The fluorescence peak was higher for the dried samples, but the peak emission wavelength remained the same. The fluorescence peak was centred at 502 nm when excited by a 405 nm laser; with the peak intensity increased with excitation energy. When excited by a pulsed 355 nm laser, two fluorescence peaks in the blue and green region were detected at 424 nm and 495 nm. The green peak shifted slightly as compared to those excited at 405 nm. The same dependence of fluorescence peak on the excitation wavelength has been reported in laser irradiation of carbon powder^20^ and micron and ns-sized diamond^36^. The dependent of the fluorescence peak on the excitation wavelength was explained to be caused by the combined effect of various types of oxygenous groups and low-lying effects of OH and C = O adsorbed on nanodiamonds^36^. It is noted that the presence of C = O also caused a small peak at ~289 eV in the XPS spectra of the sample obtained here (Fig. 4b) and in the reported work^32^.

**Figure 6:**
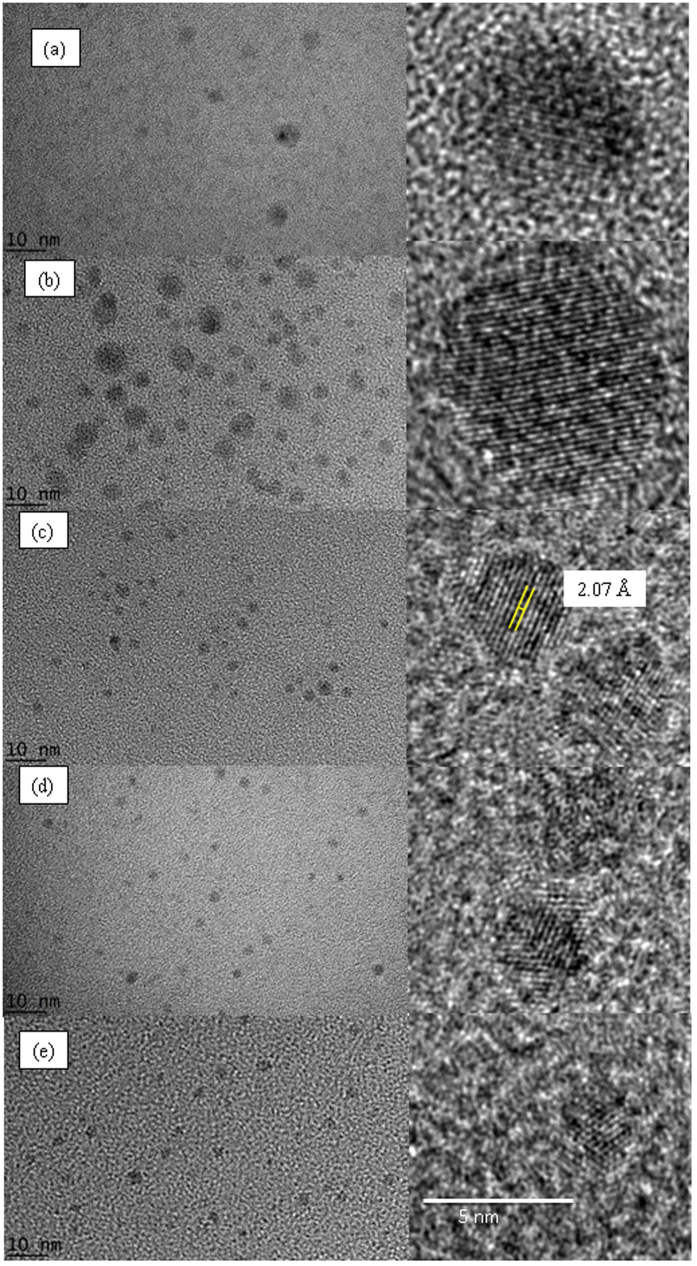
TEM of samples grown with different laser energy. (**a**) 135 μJ (region 2) for 3 hours. The samples are grown for 1 hour in (**b**) 180 μJ (region 3), (**c**) 330 μJ (region 4), (**d**) 460 μJ (region 4) and (**e**) 620 μJ (region 5).

The TEM images for the as-grown samples at different laser energy/regions are shown in Fig. 6. Fs laser irradiation was performed for 3 hours in region 2 (Fig. 6a) while the other samples were irradiated for 1 hour (Fig. 6b–e). In region 2, the nanodiamonds were 2–10 nm in size, and low in density. In region 3, more nanodiamonds were formed but the size distribution remained the same. When the incident laser energy was >300 μJ in region 4 and 5 where the absorbed energy was >75%, the nanodiamonds were uniform in size, typically <5 nm (Fig. 6c–e). All the samples contain isolated nanodiamonds dispersed in liquid. It is noted that the TEM images were taken typically >5 days after synthesis, the results indicate that the nanodiamonds were stable and there is no aggregation. The lattice spacing of the crystallites was 0.207 nm corresponds to the (111) phase of the cubic diamond.

## Discussion and Conclusion

We have shown for the first time that stable and homogeneous nanodiamonds with size of the order of 5 nm could be grown directly from liquid precursor under intense fs laser irradiation. The following summarizes what we observed. Fs laser filamentation occurred at a threshold of 100 μJ in region 2. However, laser induced breakdown in ethanol co-existed and was dominant in this laser energy range where the breakdown occurred at the geometrical focus of the laser beam. The overall formation process is inefficient that very low density of nanodiamonds was formed and the size varied from 2–10 nm. In region 3 where the incident laser energy was higher (150–300 μJ), the intensity of the supercontinuum increases; stronger laser filamentation was competing with laser induced breakdown process. Some emission spots were observed near the geometrical focus of the laser beam in addition to those occurred at the focal point. Higher plasma density was obtained and nanodiamonds were readily detected. The size of nanodiamonds ranges from 2–10 nm. In region 4, strong supercontinuum in the forward direction was observed. In addition, stable optical emission of C species and H species were also detected. The emission or plasma volume was larger than in other regions and it occurred well before the geometrical focus of the laser beam. Strong laser filamentation in ethanol occurred in this energy range and the dissociation of ethanol and formation of nanodiamonds was dominated by laser filamentation. Homogeneously distributed nanodiamonds of <5 nm were obtained. However, further increase in incident laser energy (region 5) induced direct vaporization of ethanol and plasma generation was affected. In this region, the percentage of absorbed energy increased slightly, the resultant nanodiamonds remained <5 nm but the nanodiamond density did not increase; as seen from the absorbance of the samples and TEM images.

The results show that nanodiamonds were formed in ultrafast laser irradiation of liquid precursor of carbon where the formation is dependent on the incident laser energy. The detail formation mechanisms and dynamics involved are unclear at the moment as the experimental conditions are unlike those in a typical PLAL process. The best region for homogeneous nanodiamond growth lies within region 4 where the energy absorbed by ethanol was >75%. At an incident power well beyond the critical power for self-focusing, fs laser irradiation leads to filamentation and plasma formation in ethanol. The inherent mechanism in laser filamentation- intensity clamping^37^,^38^ is believed to play an important role in the homogeneity of the size distribution of the generated particles. The phenomenon dictates that intensity is clamped inside any filament, thus the intense ultrafast laser interaction in each filament will be almost identical. This led to a naturally controlled environment for reactions for dissociation and nucleation which results in the formation of nanodiamonds with homogeneous size distribution.

We have shown a new method of nanoparticles synthesis based on ultrafast laser irradiation of liquid precursor, demonstrated by nanodiamonds formation from ethanol. The bottom-up growth may be particularly favourable for nanoparticles which has a stable phase in the nanoscale such as nanodiamond. Based on the calculated heat of formation of diamond and graphitic clusters^39^, it was concluded that at a size ~3 nm, nanodiamonds are more stable form of carbon while another calculation shows a window of stability in the range of approximately 1.9–5.2 nm^40^. Overall, the one-step process presented here is simple and yet versatile. The synthesis of other nanomaterials from liquid is possible and the process allows a wide variety of precursor or functionalized liquid to be used. In addition, intensity clamping in fs laser filamentation is beneficial in controlling the size of nanoparticles as it control the laser-materials interactions.

## Method summary

A femtosecond laser (Amplitude Systemes, s-Pulse HP) with pulse width of 500 fs, 1030 nm at 1 kHz repetition rate was used for irradiation of pure ethanol (Aldrich, 99.4%) in a quartz cell (1 cm × 1 cm × 4.5 cm). The laser beam with a beam size of 2.5 mm diameter was focused by using a lens into ethanol in the quartz cell from the top opening of a quartz cell. The height of the ethanol solution was 3 cm, corresponding to 3 ml in volume. The geometrical focus of the laser beam was 2 cm from the bottom of ethanol solution, and 1 cm from the top of the level of ethanol (Fig. 1a). The laser’s pulse energy, controlled by an acousto-optic modulator was varied between 10 μJ to 650 μJ. The percentage of energy absorbed by ethanol solution upon irradiation is obtained by measuring the incident laser energy and transmitted energy of the sample, averaging from 1000 pulses. The irradiation process was performed at different laser energy, at 1 KHz repetition rate for 60 minutes. During the process, optical emissions were measured by using a spectrometer (Avantes, Avaspec 3648, 0.3 nm resolution) in the forward direction and also in the direction perpendicular to the laser axis. The spectra were identified with reference to the atomic spectra database^21^ (Table S1). The C_2_ swan band and high pressure band are shown in Table S2^22^,^23^.

The absorption spectra of the samples after irradiations were measured by a UV-visible spectrophotometer (Avantes) with reference to the pure ethanol before irradiation. The fluorescence spectra of the samples were obtained by using 355 nm laser and a 405 nm laser as the excitation source. The sample in solution form was casted onto pre-cleaned silicon wafer and glass slide and dried for SEM, confocal Raman spectroscopy (Wiitec, 488 nm), Xray photoelectron spectroscopy and fluorescence measurement. TEM samples were prepared by dipping the Cu grid into ethanol that had been irradiated and dried for analysis.

## Additional Information

**How to cite this article**: Nee, C.-H. *et al*. Direct synthesis of nanodiamonds by femtosecond laser irradiation of ethanol. *Sci. Rep*. **6**, 33966; doi: 10.1038/srep33966 (2016).
